# Constructing college students’ musical cultural identity and self-confidence under cross-regional cultural integration: an integrative study based on cultural connections and multicultural experiences

**DOI:** 10.3389/fpsyg.2026.1674065

**Published:** 2026-01-27

**Authors:** Xiong Huijing, Hou Yunlong, Liang Yuyao, Cao Xinyi

**Affiliations:** School of Music, Jiangxi Normal University, Nanchang, Jiangxi, China

**Keywords:** cultural identity, hometown music-cultural connection, implicit cognition, multicultural experiences, music-cultural self-confidence

## Abstract

This study investigated the complex interplay between multicultural experiences (MCEs) and hometown music-cultural connection (HMC) in shaping the musical cultural self-confidence of Chinese college students. Through three studies (*N*₁ = 432, *N*₂ = 222, *N*₃ = 85), we found that while real-world MCEs were associated with lower explicit confidence, this relationship was masked by a positive indirect effect through strengthened HMC. However, this mediation was not observed when assessing implicit affective associations. Crucially, an experimental manipulation of MCEs yielded no significant effects, yet HMC consistently predicted higher confidence across both explicit and implicit cognitive measures. These results demonstrate that HMC is a stable personal resource sustaining cultural confidence, whereas the influence of MCEs is more nuanced and may not be easily induced in the short term. The findings highlight the importance of fostering deep cultural attachments for identity stability in an increasingly mobile world.

## 1 Introduction

The increasing domestic mobility of university students within China (Office of the Leading Group of the Seventh National Population Census of the State Council, 2021) presents a critical yet understudied context for understanding how individuals maintain music-cultural confidence (MCC)—the positive appraisal of one’s local musical heritage—amidst exposure to diverse regional cultures. While extensive research on cross-national adaptation has shown that broad multicultural exposure can weaken identification with one’s home culture (Lee et al., 2018; Repke and Benet-Martínez, 2017; Grove and Hansel, 1983), the psychological impact of within-country multicultural experiences (MCEs) remains largely unknown (Chen et al., 2021; Chen et al., 2019; Li et al., 2022; Berry et al., 2011). This gap is significant, as domestic cultural mobility may involve distinct processes compared to international transitions, yet its specific effect on musical cultural identity is not well understood.

The primary aim of this research is to investigate the complex interplay between MCEs, hometown music-cultural connection (HMC)—defined as the emotional bond to one’s local musical heritage—and MCC. Drawing on attachment theory (Hong et al., 2013; Keefer et al., 2014; Schatz and Lavine, 2007), we propose that HMC may serve as a compensatory resource, sustaining MCC when individuals encounter contrasting cultural contexts (Wagoner and Hogg, 2017). Furthermore, we examine these relationships at dual psychological levels: explicit (conscious) confidence and implicit (automatic) associations, which may operate independently (Wilson et al., 2000; Niu and Willoughby, 2018). The distinction is critical, as MCEs and HMC might influence these levels through different affective and cognitive pathways.

To address these gaps, we conducted three integrated studies. Study 1 employed a cross-sectional survey to establish the basic correlational relationships and test the mediating role of HMC. Study 2 incorporated the Single-Category Implicit Association Test (SC-IAT) to examine whether these relationships extend to the implicit affective level. Study 3 employed an experimental design, using an in-group preference paradigm to manipulate multicultural exposure and investigate causal effects on implicit cognitive aspects of MCC. Through this multi-method approach, we aim to move from correlation toward causation while providing a comprehensive understanding of how hometown cultural connections sustain musical cultural confidence in an increasingly mobile society.

### 1.1 Current research and hypotheses

To address these gaps, we propose the following hypotheses, which are tested through a sequence of three studies designed to move from correlation toward causation while examining both explicit and implicit processes:

*H1*: Multicultural experiences (MCEs) will negatively predict explicit music-cultural confidence (MCC).

*H2*: Hometown music-cultural connection (HMC) will positively predict both explicit and implicit MCC.

*H3*: HMC will compensate for the potential negative effects of MCEs on confidence, particularly at the explicit level.

To test these hypotheses, we employed a multi-method, sequential approach. Study 1 used a cross-sectional survey to establish basic correlations and test for the proposed mediating role of HMC. Building on this, Study 2 incorporated an implicit measure (SC-IAT) to examine whether the observed relationships extend to the automatic, unconscious level. Finally, Study 3 employed an experimental design to manipulate multicultural exposure, allowing for causal inference regarding its impact on the implicit cognitive dimensions of confidence. This integrative approach aims to provide a comprehensive understanding of how cultural connections sustain cultural confidence amidst mobility.

## 2 Study 1: correlational analysis of multicultural experiences, hometown musical cultural connections, and musical cultural confidence

Study 1 employed a cross-sectional survey design to investigate the initial relationships between multicultural experiences (MCEs), hometown music-cultural connection (HMC), and music-cultural confidence (MCC). The primary objectives were to: (1) establish the bivariate correlations among these variables, and (2) test the hypothesis that HMC mediates the relationship between MCEs and MCC.

### 2.1 Method

#### 2.1.1 Participants and sampling procedure

*A priori* sample size estimation was conducted using G*Power 3.1 (Faul et al., 2009) for a multiple regression analysis with three predictors. Setting α = 0.05 and power (1-β) = 0.80 to detect a medium effect size (*f*^2^ = 0.15), the analysis indicated a minimum required sample size of 77 participants. This effect size estimate was consistent with correlations (absolute *r* values ranging from 0.21 to 0.49) observed in prior related studies (Hong et al., 2013; Lee et al., 2018; Repke and Benet-Martínez, 2017).

An initial pool of 600 volunteers was recruited from comprehensive universities. The study received ethical approval from the Institutional Review Board of Jiangxi Normal University. All participants provided written informed consent prior to participation. During data screening, 35 participants were excluded due to inconsistent responses (e.g., providing invalid answers to open-ended questions). The final valid sample consisted of 432 participants (202 males, 230 females) with a mean age of 20.2 years (SD = 3.56). All data were anonymized upon collection by replacing identifiable information with unique participant codes, and handled in strict accordance with ethical guidelines.

#### 2.1.2 Measures

##### 2.1.2.1 Hometown music-cultural connection

Hometown music-cultural connection was assessed using the 20-item scale developed by Hong et al. (2013). Participants rated their agreement on a 7-point Likert scale (1 = strongly disagree, 7 = strongly agree). After reverse-scoring specific items, higher average scores indicated stronger attachment (i.e., lower attachment anxiety and avoidance). The scale demonstrated good internal consistency in the present sample (Cronbach’s α = 0.80).

##### 2.1.2.2 Music-cultural confidence

Music-cultural confidence was measured using a 10-item questionnaire adapted from Zhou and Bi (2020). Responses were given on a 7-point Likert scale (1 = strongly disagree, 7 = strongly agree), with higher mean scores reflecting greater levels of MCC. This measure exhibited excellent reliability (Cronbach’s α = 0.90).

##### 2.1.2.3 Multicultural experience

A composite index was created to capture geographic multicultural exposure, comprising (a) the total years of residence in cities other than their hometown and (b) the total number of cities visited. Data were winsorized to address outliers and then normalized using min-max scaling to a 0–1 range. The two normalized scores were summed to form a single MCEs index.

#### 2.1.3 Procedure

Data collection was administered online via the Qualtrics survey platform. After providing informed consent, participants first completed a demographic questionnaire. The order of the main measures (the HMC Scale, the MCC Questionnaire, and the MCEs items) was randomized across participants to control for order effects. The entire survey took approximately 15–20 min to complete.

### 2.2 Results

Descriptive statistics and intercorrelations for all study variables are presented in Table 1. Correlation analyses revealed a significant positive correlation between multicultural experiences (MCEs) and hometown music-cultural connection (HMC) (*r* = 0.28, *p* < 0.001), and a significant positive correlation between HMC and music-cultural confidence (MCC) (*r* = 0.17, *p* < 0.001). MCEs was not significantly correlated with MCC (*r* = −0.05, *p* = 0.320).

**Table 1:** Descriptive statistics and correlation coefficients for main variables (Study 1, *N* = 432).

Variable	*M*	SD	1	2	3
1. Multicultural experience	0.50	0.40	–		
2. Hometown music-cultural connection	4.53	0.72	0.28***	–	
3. Music-cultural confidence	5.09	1.00	−0.05	0.17**	–

**p < 0.01, ***p < 0.001.

A series of regression analyses were conducted to further investigate these relationships. MCEs significantly predicted HMC, β = 0.28, *t*(430) = 4.39, *p* < 0.001, Cohen’s *f*^2^ = 0.08, 95% CI [0.04, 0.15]. However, MCEs did not directly predict MCC, β = −0.06, *t*(430) = −0.75, *p* = 0.564, Cohen’s *f*^2^ < 0.01. In contrast, HMC demonstrated a significant positive predictive effect on MCC, β = 0.18, *t*(430) = 2.73, *p* = 0.007, Cohen’s *f*^2^ = 0.02, 95% CI [0.01, 0.06].

**Figure 1:**
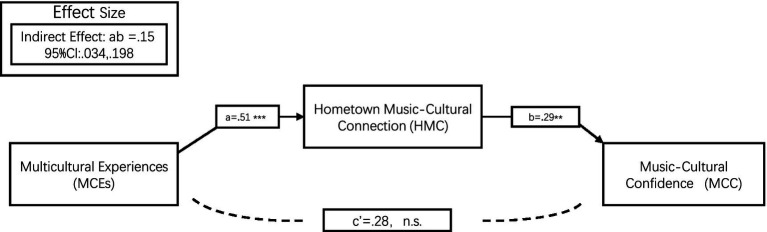
The mediating role of hometown music-cultural connection (HMC) in relationship mulicuural experiences (MCES) ansic-cultural in Study 1 (*N* = 432). Path coefficients are unstandardized. The indirect effect (ab) was significant, as shown by its 95% bias-corrected bootstrap confidence interval which does not include zero. The direct effect (c’) was non-significant. **p < 0.001, *p* < 0.01, n.s. = not significant.

Given that the direct effect of MCEs on MCC (path c = −0.13, *p* > 0.05) was opposite in direction to its indirect effect through HMC, we tested for a masking effect using mediation analysis with the PROCESS macro (Model 4, Wen and Ye, 2014). The results indicated significant path coefficients for both a (MCEs → HMC; *a* = 0.51, *p* < 0.001) and b (HMC → MCC; *b* = 0.29, *p* < 0.01), while the direct path c’ remained non-significant (*c*’ = −0.28, *p* > 0.05). The indirect effect (ab) was statistically significant, with a point estimate of 0.15 and a 95% bias-corrected bootstrap confidence interval based on 5,000 samples not including zero [0.034, 0.198]. As ab was opposite in sign to c’, a masking effect was confirmed (see Figure 1). The absolute value of the ratio of the indirect to direct effect was |52.8%|, indicating that the indirect effect played a substantial role in the overall relationship.

### 2.3 Discussion

The findings from Study 1 support the hypothesized mediating role of HMC between MCEs and MCC, revealing a significant masking effect. Specifically, multicultural experience positively predicted stronger hometown music-cultural connections (Kosmitzki, 1996). This suggests that individuals may strengthen emotional ties to their hometown culture as a potential way of mitigating acculturative stress encountered in new environments. Furthermore, hometown music-cultural connections positively predicted music-cultural confidence, consistent with theories emphasizing the role of cultural attachment in fostering positive identity formation.

Notably, the analysis revealed that while the direct effect of MCEs on MCC was non-significant, its indirect effect through HMC was significant and positive. This pattern indicates that MCEs may simultaneously exert a weak negative direct influence on confidence while strengthening it indirectly through enhanced cultural connections—a finding that helps reconcile previous reports of multicultural experiences potentially weakening cultural identity (Lee et al., 2018; Repke and Benet-Martínez, 2017).

However, these conclusions are based solely on explicit self-report measures, which cannot capture unconscious processes and may be influenced by social desirability. To address this limitation and examine whether similar relationships operate at the implicit level, Study 2 incorporates an implicit association test (SC-IAT) to provide a more comprehensive understanding of the psychological mechanisms underlying music-cultural confidence.

## 3 Study 2: examining the relationships at the implicit level

Building on the findings of Study 1, Study 2 aimed to investigate whether the relationships between multicultural experiences (MCEs), hometown music-cultural connection (HMC), and music-cultural confidence (MCC) extend to the implicit level. A primary limitation of Study 1 was its reliance on explicit self-report measures, which are susceptible to social desirability biases and may not capture automatic, unconscious associations. To address this, we incorporated the Single-Category Implicit Association Test (SC-IAT) to measure implicit music-cultural confidence (IMCC). We hypothesized that the negative association between MCEs and MCC, as well as the positive association between HMC and MCC, would be evident at the explicit level. However, given the potential dissociation between explicit and implicit attitudes, we explored whether these relationships would manifest differently for implicit confidence.

### 3.1 Method

#### 3.1.1 Participants

An *a priori* power analysis was conducted using G*Power 3.1 (Faul et al., 2009) for a multiple regression analysis with three predictors. Setting α = 0.05 and power (1-β) = 0.80 to detect a medium effect size (*f*^2^ = 0.15), the analysis indicated a minimum required sample size of 77 participants. This estimate was consistent with effect sizes observed in prior related research (Hong et al., 2013). A total of 222 university students were initially recruited.

The study received ethical approval from the Institutional Review Board of Jiangxi Normal University. All participants provided written informed consent prior to their participation. Data from 3 participants who did not complete the critical implicit measure (SC-IAT) were excluded, resulting in a final valid sample of 219 participants (89 males, 130 females), with ages ranging from 17 to 29 years (*M* = 20.66, SD = 1.93). All data were anonymized immediately after collection by replacing identifiable information with unique participant codes and were handled in strict accordance with the ethical guidelines approved by the Institutional Review Board. The final sample size of 219 provided sufficient statistical power (>99%) to detect the hypothesized medium effects.

#### 3.1.2 Measures

##### 3.1.2.1 Implicit music-cultural confidence

The Single-Category Implicit Association Test (SC-IAT; Karpinski and Steinman, 2006) was employed to assess implicit attitudes toward hometown music culture. The task consisted of a practice block followed by critical test blocks. Participants categorized stimuli representing “hometown music culture,” positive words, and negative words using designated key presses. The D-score was calculated following the standard algorithm (Charlesworth and Banaji, 2019). Reaction times below 350 ms or above 1,500 ms were recoded to these thresholds. Error trials were replaced with the block mean for correct trials plus a 400-ms penalty. The D-score was derived by dividing the difference in mean reaction times between the incompatible and compatible tasks by the pooled standard deviation of all correct reaction times. Higher D-scores indicate more positive implicit associations with hometown music culture. The SC-IAT demonstrated good internal consistency in this study, with a Spearman-Brown corrected split-half reliability coefficient of 0.78.

##### 3.1.2.2 Explicit measures

The same self-report scales from Study 1 were used to assess MCEs, HMC (Cronbach’s α = 0.76), and explicit MCC (Cronbach’s α = 0.87). Demographic variables were collected identically to Study 1.

#### 3.1.3 Procedure

Participants completed the assessments in a fixed sequence: (1) the SC-IAT, (2) the Music Culture Attachment Questionnaire, (3) the Music Culture Confidence Questionnaire, and (4) the multicultural experience and demographic items. The fixed order was implemented to prioritize the assessment of implicit attitudes before any explicit questioning, thereby minimizing the potential for conscious reflection to influence automatic responses. While this fixed order may introduce order effects, it was deemed necessary to ensure the validity of the implicit measure, which was the primary focus of Study 2.

### 3.2 Results

Descriptive statistics and intercorrelations for all variables in Study 2 are summarized in Table 2. Correlation analyses revealed a significant negative correlation between multicultural experiences (MCEs) and explicit music-cultural confidence (MCC) (*r* = −0.21, *p* < 0.05), and a significant positive correlation between hometown music-cultural connection (HMC) and explicit MCC (*r* = 0.21, *p* < 0.01). However, implicit music-cultural confidence (IMCC) was not significantly correlated with MCEs (*r* = 0.04, *p* > 0.05), HMC (*r* = −0.05, *p* > 0.05), or explicit MCC (*r* = 0.05, *p* > 0.05).

**Table 2:** Descriptive statistics and correlation coefficients for main variables (Study 2, *N* = 219).

Variable	*M*	SD	1	2	3	4
1. Multicultural Experience (MCE)	0.53	0.37	–			
2. Hometown Connection (HMC)	4.52	0.52	−0.01	–		
3. Explicit Confidence (MCC)	5.33	0.82	−0.21*	0.21**	–	
4. Implicit Confidence (IMCC)	0.16	0.25	0.04	−0.05	−0.05	–

*p < 0.05, **p < 0.01.

A series of regression analyses were conducted to further examine these relationships. MCEs did not significantly predict HMC, β = −0.01, *t*(217) = −0.07, *p* = 0.944, Cohen’s *f*^2^ < 0.01. In contrast, MCEs significantly negatively predicted explicit MCC, β = −0.21, *t*(217) = −2.59, *p* = 0.010, Cohen’s *f*^2^ = 0.03, 95% CI [0.01, 0.08]. Concurrently, HMC was a significant positive predictor of explicit MCC, β = 0.21, *t*(217) = 2.62, *p* = 0.009, Cohen’s *f*^2^ = 0.03, 95% CI [0.01, 0.08].

To test the hypothesized mediating role of HMC, a mediation analysis was performed using the PROCESS macro (Model 4). The path coefficient for MCEs on HMC (path a) was not significant, *a* = −0.01, *t*(217) = −0.07, *p* > 0.05, Cohen’s *f*^2^ < 0.01. The path from HMC to explicit MCC (path b) was significant, *b* = 0.21, *t*(217) = 2.62, *p* < 0.01, Cohen’s *f*^2^ = 0.03, 95% CI [0.01, 0.08]. The direct effect of MCEs on explicit MCC (path c’) was significant, *c*’ = −0.21, *t*(217) = −2.59, *p* < 0.05, Cohen’s *f*^2^ = 0.03, 95% CI [0.01, 0.08]. The indirect effect (ab*) was not significant, with a point estimate of −0.002 and a 95% bias-corrected bootstrap confidence interval based on 5,000 samples that included zero [−0.046, 0.026]. Therefore, no mediating effect was found.

Parallel analyses using the IMCC D-score yielded null results: neither MCEs (β = 0.04, *p* > 0.05, Cohen’s *f*^2^ < 0.01) nor HMC (β = −0.05, *p* > 0.05, Cohen’s *f*^2^ < 0.01) significantly predicted IMCC, and consequently, no mediation effect was observed.

### 3.3 Discussion

The results of Study 2 present a clear dissociation between explicit and implicit measures. At the explicit level, we replicated the key finding from Study 1: MCEs negatively predicted explicit MCC, while HMC positively predicted it. However, the hypothesized mediating role of HMC was not supported in this sample, as MCEs did not significantly predict HMC levels, highlighting the potential variability in these relationships across different contexts.

Most notably, the findings at the implicit level were distinct. Neither MCEs nor HMC showed any significant association with IMCC. This clear dissociation is consistent with dual-attitude models (Wilson et al., 2000; Briñol et al., 2006; Cunningham and Wigfall, 2020), which posit that implicit and explicit attitudes can represent separate psychological constructs with distinct antecedents and consequences. The SC-IAT used in this study primarily captures the affective component of implicit attitudes (i.e., automatic positive or negative evaluations). The null findings suggest that the affective associations tapped by the SC-IAT operate independently of the more cognitive-evaluative processes measured by the explicit scales and influenced by MCEs and HMC (Muschalik et al., 2019). This points to a critical limitation of our measurement approach for capturing the full spectrum of implicit music-cultural confidence, which may also encompass cognitive facets such as automatic associations with cultural superiority or resilience.

These findings raise an important theoretical question: if the affective-implicit dimension is distinct, how is confidence manifested at the implicit-cognitive level? To address this question and to move beyond correlational designs to establish causal direction, Study 3 employed an experimental design. By actively manipulating multicultural exposure and utilizing an in-group preference paradigm (Enock et al., 2018; Smith and Henry, 1996) designed to assess cognitive biases, we aimed to isolate the causal effect of MCEs on the implicit cognitive dimension of music-cultural confidence.

## 4 Study 3: experimental manipulation of multicultural experiences and causal inference

Study 3 employed an experimental design to investigate the causal effects of multicultural experiences (MCEs) on hometown music-cultural connection (HMC) and music-cultural confidence (MCC). While Studies 1 and 2 revealed correlational patterns, they could not establish causality. Furthermore, the null findings at the implicit level in Study 2 suggested the need for a paradigm that captures cognitive, rather than purely affective, aspects of implicit cognition. Therefore, Study 3 had two primary aims: (1) to test whether an acute laboratory manipulation of MCEs causes changes in HMC and MCC, and (2) to utilize an in-group preference paradigm, which assesses cognitive processing biases, to measure implicit music-cultural confidence (IMCC). We hypothesized that if real-world MCEs effects on confidence can be simulated in the laboratory, participants in the multi-music culture condition would show lower explicit MCC and altered implicit cognitive biases compared to those in the mono-culture condition. Alternatively, based on the stability of individual differences observed in the previous studies, we expected that HMC would remain a robust predictor of both explicit and implicit outcomes regardless of the experimental manipulation.

### 4.1 Method

#### 4.1.1 Participants

An *a priori* power analysis was conducted using G*Power 3.1 (Faul et al., 2009) for an analysis of variance (ANOVA). Setting α = 0.05 and power (1-β) = 0.80 to detect a medium effect size (*f* = 0.25), the analysis indicated a minimum required sample size of 81 participants. This effect size estimate is consistent with experimental manipulations in related social cognition research (Enock et al., 2018).

A total of 88 university students were initially recruited. The study received full ethical review and approval from the Institutional Review Board of Jiangxi Normal University. All participants provided written informed consent prior to their enrollment in the experiment. Following screening, three participants who had previously resided in Tianjin or Guangzhou for over 1 month were excluded to control for prior extensive exposure to the target cultures. The final valid sample consisted of 85 participants (71 females, 14 males) with a mean age of 19.36 years (SD = 1.31). All data were anonymized immediately after collection by replacing any identifiable information with unique participant codes, and were handled in strict accordance with the ethical guidelines approved by the Institutional Review Board.

Participants were randomly assigned to either the mono-music culture group (*n* = 41) or the multi-music culture group (*n* = 44). The final sample size of 85 provided sufficient statistical power (>80%) to detect the hypothesized medium effect.

#### 4.1.2 Materials and measures

##### 4.1.2.1 Multicultural experience induction and manipulation check

Participants were randomly assigned to one of two conditions. The mono-music culture group read neutral materials about computer technology. The multi-music culture group read detailed descriptions of Tianjin and Guangzhou, covering their history, economy, culture, education, and cuisine, with emphasis on their distinct musical traditions (e.g., Tianjin’s folk music vs. Guangzhou’s Cantonese Opera). To enhance engagement, participants in the multi-music culture group were required to write at least 20 words associating the two cities with their musical cultures.

Crucially, to address the limitation of a purely procedural check, a quantitative manipulation check was administered immediately after the reading task. Participants rated their agreement with two statements on a 7-point Likert scale (1 = Strongly Disagree, 7 = Strongly Agree): “The materials exposed me to diverse regional cultures” and “I can perceive differences in musical culture between the cities described.” This provided a psychometric validation of the manipulation’s effectiveness.

##### 4.1.2.2 Implicit music-cultural confidence

An in-group preference paradigm (Enock et al., 2018) was adapted to assess implicit cognitive aspects of MCC. The task used abstract graphs representing in-group (supportive) and out-group (competitively opposed) music teams (Ingleby, 1967; Macmillan and Creelman, 2005). Participants’ performance was evaluated using signal detection theory metrics—the discrimination index d’ (sensitivity) and the response criterion C (bias)—alongside response times (RT) and accuracy, to assess cognitive biases toward hometown music culture.

##### 4.1.2.3 Explicit measures

The same self-report scales from Study 1 were used to measure HMC (Cronbach’s α = 0.71) and explicit MCC (Cronbach’s α = 0.86).

#### 4.1.3 Procedure

Participants completed the study in a fixed sequence under controlled laboratory conditions: (1) multicultural experience induction, (2) quantitative manipulation check, (3) implicit MCC task (in-group preference paradigm), (4) explicit questionnaires (HMC and MCC), and (5) demographic information. This sequence ensured that any effects on subsequent measures could be attributed to the experimental manipulation.

### 4.2 Results

#### 4.2.1 Manipulation check

An independent-samples *t*-test conducted on the quantitative manipulation check scores confirmed a significant difference between the multi-music culture group (*M* = 5.8, SD = 0.9) and the mono-culture group (*M* = 2.3, SD = 1.1), *t*(83) = 8.92, *p* < 0.001, Cohen’s d = 1.94, 95% CI [1.45, 2.41]. This result verifies that the experimental manipulation successfully induced differing levels of perceived multicultural exposure.

#### 4.2.2 Effects of experimental manipulation on primary outcomes

Contrary to the correlational findings of Studies 1 and 2, the central experimental tests revealed no significant effects of the multicultural experience manipulation on any of the primary outcome measures. Independent-samples t-tests showed no significant difference between the mono- and multi-cultural groups on hometown music-cultural connection, *t*(83) = −0.30, *p* = 0.765, Cohen’s d = −0.07, 95% CI [−0.52, 0.39]; or on explicit music-cultural confidence, *t*(83) = 0.54, *p* = 0.591, Cohen’s d = 0.12, 95% CI [−0.31, 0.55]. Similarly, no significant group differences were found on any of the four implicit task metrics (all *p*s > 0.05). The null findings were further supported by Bayesian analyses, which provided moderate evidence (BF₀₁ > 3) in favor of the null hypothesis for these group comparisons.

#### 4.2.3 Correlational analyses across the entire sample

Despite the null effect of the experimental manipulation, correlational analyses conducted across the entire sample (*N* = 85) replicated the core finding observed in the previous studies. Hometown music-cultural connection consistently predicted both higher explicit MCC, β = 0.39, *t*(83) = 2.52, *p* = 0.014, Cohen’s *f*^2^ = 0.08, 95% CI [0.02, 0.20], and advantages on implicit cognitive tasks. For instance, higher HMC was associated with significantly greater accuracy for hometown-related stimuli, β = 0.04, *t*(83) = 2.10, *p* = 0.039, Cohen’s *f*^2^ = 0.05, 95% CI [0.00, 0.15] (Sui and Humphreys, 2017). These relationships held after controlling for baseline confidence.

### 4.3 Discussion

The results of Study 3 provide critical boundary conditions for our theoretical model. Most importantly, the experimental manipulation of multicultural exposure—despite being successfully perceived by participants—did not yield significant effects on either hometown connection or cultural confidence. This key divergence from the correlational patterns observed in Studies 1 and 2 suggests a crucial theoretical distinction: the psychological impact of real-world, long-term MCEs may not be readily captured by a short-term, laboratory-based manipulation.

The effects of actual geographic mobility likely operate through slower, developmental pathways involving deep social comparison and sustained identity negotiation—complex processes that a brief reading task cannot fully activate (Whelan and Zelenski, 2012). Therefore, the null finding from our experiment is not an indicator of failure but an important empirical discovery. It clarifies that the phenomenon under investigation is not easily malleable in the short term and underscores that the correlational relationships identified earlier may be driven by long-term, developmental processes rather than acute situational exposures.

The robust correlational finding across the entire sample, however, further underscores the stability of individual differences in HMC. Regardless of the experimental condition, participants with stronger emotional ties to their hometown music culture exhibited higher levels of both explicit confidence and implicit cognitive advantages. This reinforces the notion that HMC is a relatively enduring personal resource, the influence of which is evident even in the absence of an experimental effect (Hobbs et al., 2023; Verplanken and Sui, 2019).

Theoretical integration of these findings suggests a more nuanced model: while real-world MCEs may influence cultural confidence through complex, long-term pathways, HMC appears to be a more direct and stable contributor that bolsters confidence across both explicit and implicit-cognitive domains. This set of results establishes a clear boundary condition for our theory, highlighting the necessity for future research to employ more potent, ecologically valid interventions (e.g., longitudinal designs or immersive virtual reality experiences) to adequately test the causal mechanisms linking multicultural experiences and cultural confidence.

## 5 General discussion

This research undertook a multi-method investigation to unravel the complex interplay between multicultural experiences (MCEs), hometown music-cultural connection (HMC), and music-cultural confidence (MCC) among Chinese college students. The findings paint a nuanced picture: while HMC consistently emerged as a robust positive contributor to MCC across explicit and implicit-cognitive domains, the role of MCEs proved to be more complex and context-dependent. The following discussion integrates these findings, critically addresses the theoretical implications of key results—especially the null findings—and outlines the limitations and pathways for future research.

### 5.1 Theoretical integration and interpretation of key findings

The most stable and compelling finding across all three studies is the positive association between HMC and MCC (Moise et al., 2019). This aligns robustly with attachment theory (Hong et al., 2013; Hossain and Lamb, 2020; Park et al., 2021; Snowshoe et al., 2017), suggesting that a secure emotional bond with one’s hometown culture serves as a foundational psychological resource that fosters a sense of security and positive identity coherence (Yap et al., 2017).

The relationship involving MCEs, however, requires a more delicate interpretation. The correlational findings of Studies 1 and 2 suggest that in real-world contexts, MCEs may present a dual effect. However, the critical null result from the experimental manipulation in Study 3 compels a re-evaluation of causal inferences. The fact that an acute laboratory induction of MCEs failed to replicate the correlational patterns sets a crucial boundary condition for our initial theoretical model. It suggests that the psychological impact of real-world MCEs is not merely a product of exposure but likely involves slower, more complex developmental or ecological processes, such as long-term identity negotiation and the accumulation of lived experiences. This key divergence challenges simple causal narratives and highlights a fundamental distinction between the correlates of long-term real-world experiences and the effects of short-term experimental manipulations.

Furthermore, the clear dissociation between explicit and implicit measures in Study 2 underscores the multifaceted nature of cultural confidence. The null findings with the SC-IAT, which taps affective associations, contrasted with the significant findings on the explicit measures and the implicit cognitive measures in Study 3, suggest that MCEs and HMC may primarily influence cognitive-evaluative components. The affective component of implicit attitudes may be more deeply ingrained and less susceptible to modification through the experiences measured here. These differential findings across measures and methodologies caution against overgeneralization and emphasize the importance of specifying the attitudinal component and measurement approach when interpreting results in this domain.

### 5.2 Critical reflection on limitations and future directions

This research has several limitations that qualify the interpretation of our findings and chart a course for future studies. First, the cross-sectional nature of Studies 1 and 2 precludes definitive causal claims. While Study 3 attempted to address causality, the lack of a potent, ecologically valid manipulation remains a significant constraint. The short-term reading task, though quantitatively verified, cannot capture the richness and emotional weight of actual multicultural living. Future research should employ more powerful interventions, such as longitudinal studies tracking students during study-abroad programs within their own country or immersive virtual reality simulations, to better test causal pathways.

Second, our measurement approach has inherent limitations. The sensitivity of the SC-IAT in this context is questionable, as it may not have fully captured the construct of implicit cultural confidence. Similarly, while the in-group preference paradigm showed validity for cognitive biases, it may not represent the affective dimensions. Future studies would benefit from employing a multi-method battery of implicit measures to triangulate the construct.

Third, the generalizability of our findings is limited by the homogeneous sample of Chinese university students (Cheung et al., 2011). Cultural confidence processes may differ significantly across populations with varying cultural backgrounds, mobility histories, and age ranges. Replication in more diverse samples is essential.

Finally, the theoretical meaning of the null experimental result must be explored further. Does it imply that MCEs do not cause changes in confidence, or that our current methodologies are insufficient to capture the causal mechanisms? Future work should directly investigate the psychological processes that mediate the long-term impact of MCEs, such as changes in social network composition, identity integration complexity (Roccas and Brewer, 2002), or perceived cultural threat (Trope and Liberman, 2010).

### 5.3 Conclusion

In conclusion, this integrative series of studies demonstrates that emotional ties to one’s hometown musical culture are a potent and consistent source of cultural confidence. In contrast, the effects of multicultural experiences are more nuanced and may operate through long-term, developmental pathways rather than acute situational exposures. The divergence between our correlational and experimental findings, far from being a weakness, provides a critical insight: the confidence-sustaining function of HMC is a stable individual-difference resource, while the effect of MCEs is complex and not easily induced in the short term. These findings suggest that educational and cultural initiatives aimed at fostering cultural confidence should prioritize nurturing deep, meaningful connections to local cultural heritage. Simultaneously, preparing individuals for multicultural encounters may be most effective when focused on long-term adaptation and identity integration strategies, rather than expecting immediate changes from brief exposures.

## Data Availability

The original contributions presented in the study are included in the article/supplementary material, further inquiries can be directed to the corresponding author.
